# Bleeding events in people with hemophilia B on prophylactic factor IX treatment: a systematic review and meta-analysis

**DOI:** 10.1016/j.rpth.2026.106954

**Published:** 2026-09-09

**Authors:** Kristina Lindsley, Arianna Nevo, Carmine Colavecchia, Lisa J. Wilcox, Priya Patel, Carlos Estevez, Cynthia Khanji, Jennifer Uyei, Steven W. Pipe, Guy Young

**Affiliations:** 1 Evidence Synthesis, IQVIA, Falls Church, Virginia, USA; 2 Evidence Synthesis, IQVIA, San Mateo, California, USA; 3 Pfizer, New York City, New York, USA; 4 Pfizer, Montreal, Quebec, Canada; 5 Pfizer, Collegeville, Pennsylvania, USA; 6 Departments of Pediatrics and Pathology, University of Michigan, Ann Arbor, Michigan, USA; 7 Hemostasis and Thrombosis Center, Cancer and Blood Disease Institute, Children’s Hospital Los Angeles, Los Angeles, California, USA

**Keywords:** bleeding, factor IX, hemophilia B, meta-analysis, systematic review

## Abstract

**Background:**

Hemophilia B is a rare, X-linked disorder leading to deficient or dysfunctional clotting factor IX and bleeding complications.

**Objectives:**

The objective of this systematic review and meta-analysis was to summarize bleeding events in people with hemophilia B of any severity without inhibitors receiving prophylactic factor IX replacement therapies to better understand disease burden and unmet needs.

**Methods:**

A systematic search was conducted to identify clinical trials and observational studies reporting annualized bleeding rate (ABR), annualized joint bleeding rate, spontaneous bleeding events, traumatic bleeding events, and percentage of patients with zero-bleeding events. Bleeding outcomes were specified as total, treated, or unspecified and analyzed using the random-effects model. Subgroup meta-analyses assessed differences by age, treatment type, follow-up duration, and publication date, when feasible.

**Results:**

The search yielded 5684 citations; 24 clinical trials and 53 observational studies were included in the meta-analysis. Treated ABR was 2.23 (95% CI, 1.52-3.27) in 13 trials; treated or unspecified ABR was 2.25 (95% CI, 1.67-3.03) in 22 trials. In 48 observational studies, treated or unspecified ABR was 2.08 (95% CI, 1.62-2.66). ABR was generally lower for patients <12 years of age (vs ≥12 years of age) and patients treated with extended half-life (vs standard half-life).

**Conclusion:**

Results from this meta-analysis reflect advances in hemophilia B treatment, but also highlight unmet needs, with many patients experiencing ≥2bleeding events per year. Inconsistent ABR definitions and variability in follow-up times may introduce heterogeneity in the combined estimates.

## Essentials

• Bleeding can still occur in people with hemophilia B receiving preventive treatment.
• We combined results from 105 studies of preventive factor IX therapies.
• Patients experienced about 2 bleeding events per year on average.
• Studies define bleeding rates differently. Standardization may improve treatment decision-making.

## 1 Introduction

Hemophilia B is a rare, inherited disease caused by defects in the *F9* gene (encoding factor [F]IX) on the X-chromosome. It leads to dysfunction or deficiency in clotting FIX [1]. Hemophilia has mild, moderate, and severe forms; the most severe forms (characterized by FIX activity < 1 IU/dL or <1% of normal) [2] are associated with spontaneous and recurrent bleeding episodes into joints and other tissues, irreversible bone and cartilage damage, chronic pain, and crippling hemophilic arthropathy [1,3,4].

Effective management of hemophilia B is crucial to prevent recurrent bleeding, complications, and to improve quality of life [4]. The World Federation of Hemophilia guidelines identify early initiation of individualized prophylaxis as the standard of care to prevent bleeding and preserve joint health [2]. In 2022, international consensus recommendations for treatment of severe hemophilia B advised that prophylactic administration of either plasma-derived or recombinant FIX and either standard half-life (SHL) or extended half-life (EHL) recombinant FIX may be considered [5]. Similarly, in 2024, the International Society on Thrombosis and Haemostasis strongly recommends prophylactic FIX therapy over episodic treatment for patients with severe hemophilia B without inhibitors [6].

Since these recommendations, additional nonfactor prophylactic treatments, such as inhibitors of natural anticoagulants (marstacimab, concizumab, and fitusiran), and gene therapy (etranacogene dezaparvovec-drlb), have been approved in the United States for patients ≥6 years (marstacimab) [7], ≥12 years, and adults, respectively [4,5,[8], [9], [10]]. As research continues, the treatment landscape for hemophilia B is likely to see further advancements aimed at improving outcomes and quality of life.

Clinical trials and observational studies are essential for evaluating the effectiveness of new therapies for hemophilia B. Key effect measures typically include annualized bleeding rate (ABR) and the percentage of patients without bleeding events. ABR measures the average number of bleeding episodes a patient experiences over a set period. ABR may be further defined by the type (eg, total, treated, spontaneous) and location (eg, joint) of bleeding episodes [11]. These metrics serve as proxies for a therapy’s ability to reduce bleeding frequency, with lower ABR indicating better control of bleeding. Understanding potential differences in bleeding events between clinical trials and real-world studies may offer additional perspective on treatment effectiveness.

The purpose of this systematic review and meta-analysis was to estimate bleeding events in patients with hemophilia B of any severity without inhibitors, treated with prophylactic factor replacement therapies within clinical trials and observational studies.

## **2** Methods

The methods for this systematic review follow those published in the Cochrane Handbook for Systematic Reviews of Interventions [12] and the protocol was registered prospectively in PROSPERO (registration ID CRD420251057762). The Preferred Reporting Items for Systematic Reviews and Meta-Analyses (PRISMA) checklist [13] is available in Supplementary Table 1.

### 2.1 Eligibility criteria

Study eligibility criteria were defined in terms of population, interventions, comparisons, outcomes, and study design (PICOS). Both interventional and observational studies of patients with hemophilia B of any severity without inhibitors receiving prophylactic standard or EHL factor IX replacement therapies were included (Table).

**Table:** PICOS eligibility criteria.

PICOS	Study eligibility criteria
**Population**	• Patients with inherited hemophilia B without active FIX inhibitors
**Interventions**	• Prophylaxis FIX replacement treatment • Lead-in phase from trials of gene therapy and rebalancing agents, in which active treatment with FIX prophylaxis was assessed
**Comparators**	• Any of the above interventions or none (no comparison is required)
**Outcomes**	At least one bleeding event outcome: • Annualized bleeding rate • Annualized joint bleeding rate • Spontaneous bleeding events • Traumatic bleeding events • Percentage of participants with zero-bleeding events
**Study design**	• Interventional studies including clinical trials (RCTs, single-arm trials, lead-in, and non-RCTs) and extension studies • Observational studies including registry studies, claims database studies, cohort studies, cross-sectional studies, case-control studies
**Other limits**	• Only English-language articles

FIX, factor IX; PICOS, population, interventions, comparisons, outcomes, and study design; RCT, randomized controlled trial.

### 2.2 Study identification

A comprehensive literature search using multiple data sources was conducted to identify relevant published studies. Bibliographic databases, clinical trial registries, conferences and congresses, and references of reviews were searched. Systematic searches were conducted using the OvidSP platform (MEDLINE and Epub Ahead of Print, In-Process, In-Data-Review and Other Nonindexed Citations and Daily; EMBASE; and Cochrane Central Register of Controlled Trials) and clinical trial registers (ClinicalTrials.gov and the World Health Organization International Clinical Trials Registry Platform).

Search strategies were developed based on keywords and appropriate indexing terms for hemophilia B and eligible study designs in collaboration with a medical librarian. Searches were limited to English-language records. No date restriction was employed. The complete search strategy is provided in Supplementary Table 2.

### 2.3 Study selection and data collection

All records identified through the systematic review were managed using EndNote and imported into DistillerSR for screening. A two-step process was used. First, titles and abstracts were screened by an experienced reviewer and quality-checked by a second reviewer. DistillerSR’s artificial intelligence technology was used to rescreen all excluded records, and those with a probability ranking >85% were rescreened by a third reviewer. Second, full texts were independently screened by 2 reviewers, with disagreements adjudicated by a third reviewer. Studies meeting the population, interventions, comparisons, outcomes, and study design criteria were included for data extraction. Data were extracted using a standardized template capturing study and patient characteristics, treatments, and outcomes. Two reviewers (AN and KL) independently extracted data, with discrepancies resolved by a third reviewer (JU).

### 2.4 Outcomes

Bleeding events were defined as total (treated and untreated), treated, or unspecified. ABR definitions were classified as unspecified when they described annualizing bleeding events over the observation or efficacy period, but did not indicate whether the numerator included treated bleeds only or both treated and untreated bleeds. For example, studies were considered unspecified when ABR was defined as the number of reported bleeding events divided by months of observation and multiplied by 12 [14], or as the number of bleeding episodes per participant divided by days in the efficacy period and multiplied by 365.25 [15]. Bleeding events were analyzed as rate or binary outcomes:

• Rate outcomes included: ABR (specified as total ABR, treated ABR, or treated and unspecified ABR), annualized joint bleeding rate (AJBR; specified as total AJBR, treated AJBR, or treated and unspecified AJBR), spontaneous bleeding events, and traumatic bleeding events.
• Binary outcomes included: proportion of patients with zero-bleeding events (specified as total, treated, or treated and unspecified).

### 2.5 Feasibility assessment

A feasibility assessment was conducted to determine whether a meta-analysis could be appropriately performed based on the evidence identified through the systematic literature review. This assessment evaluated the consistency and completeness of outcome reporting across studies, the comparability of study designs and patient populations, and the presence of potential effect modifiers. Assumptions required for analysis were documented, and subgroup analyses were proposed where heterogeneity was anticipated. Studies were not eligible for meta-analysis for the following reasons: (1) did not report eligible outcome data; (2) only reported mean or median with no variance for rate outcomes; (3) outcome measurements not comparable; (4) did not report the number of patients; or (5) data were not stratified for patients receiving prophylaxis vs on-demand therapy. Based on the feasibility assessment, subgroup analyses were conducted to investigate differences based on the following factors: age (<12 years vs ≥12 years), treatment type (EHL or SHL), follow-up duration (<12 months vs ≥12 months), and year of publication (before 2020 vs 2020 and after).

### 2.6 Statistical analysis

Meta-analyses were conducted using a two-stage approach: (1) transformed summary estimates were derived for each study, and (2) estimates were pooled across studies and back-transformed to the original scale. For ABR outcomes, log-transformed rates and SEs were calculated; pooled estimates were exponentiated to return to the natural scale. The log(rate) was calculated as the logarithm of the reported mean. When SEs were missing, they were estimated from the 95% CIs using the formula: [log(upper 95% limit) − log(lower 95% limit)]/3.92. If 95% CIs were unavailable, SDs of the log(rate) were estimated via the delta method, and SE was computed as: SD(log(rate))/sqrt(*n*). When mean and/or SD were not reported, they were imputed from the median and IQR or range using the methods described by Wan et al. [16].

For studies reporting zero-bleeding events, the Freeman–Tukey double arcsine transformation was applied in its original formulation [[17], [18], [19]]. Back-transformation of pooled estimates was performed using inverse variance methods as described by Doi and Xu [17]. For studies reporting bleeding rate outcomes for multiple groups (eg, by subgroup or treatment arm), a fixed-effect meta-analysis was used to derive a single treatment estimate for each study, such that each study is only represented once for each analysis. Similarly, event counts were aggregated for zero-bleeding outcomes to derive a single treatment estimate for each study. When pooling across studies, both random-effects models (restricted maximum likelihood) and fixed-effect models were implemented. Forest plots were generated to visualize overall and subgroup results. Heterogeneity was assessed using the Cochrane Q test (*P* value), I^2^ statistic, τ^2^ (between-study variance), and 95% prediction intervals.

Publication bias was assessed using funnel plots and Egger’s test when ≥10 studies were included. Egger’s test evaluates funnel plot symmetry; a *P* value < .05 indicates asymmetry and potential bias.

## 3 Results

### 3.1 Included studies

Database searches identified 5684 records as of January 29, 2025. Following deduplication, 4,553 unique records underwent title and abstract screening, of which 413 were retained for full-text review. A total of 194 records meeting the eligibility criteria were included after full-text review, and 50 additional records were identified from searches of review bibliographies, conference proceedings, and trial registries. Overall, 105 studies (from 244 reports) were included in the review. The inclusion and exclusion of studies are summarized in the Preferred Reporting Items for Systematic Reviews and Meta-Analyses flow diagram (Figure 1).

**Figure 1:**
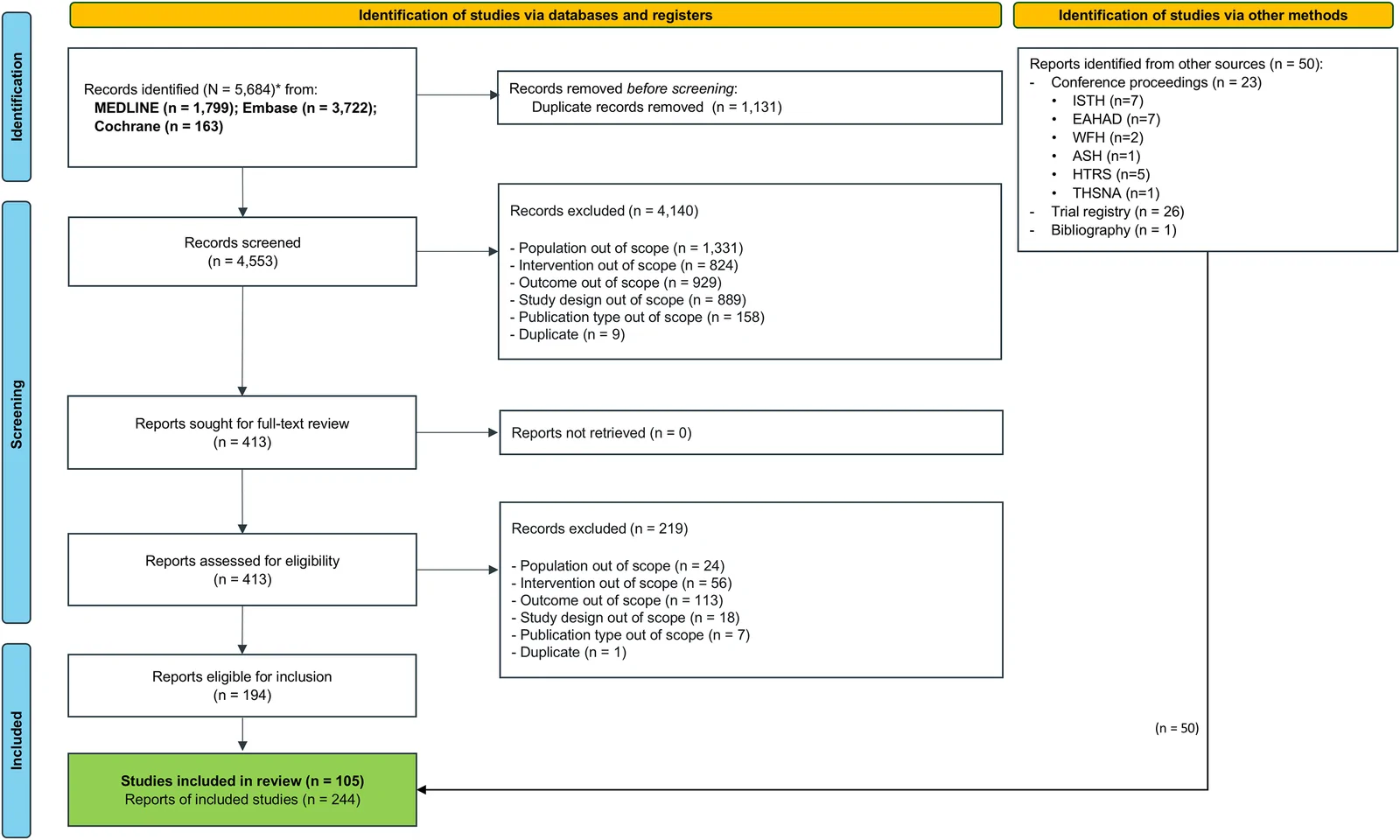
Preferred Reporting Items for Systematic Reviews and Meta-Analyses diagram. ASH, American Society of Hematology; EAHAD, European Association for Haemophilia and Allied Disorders; HTRS, Hemostasis and Thrombosis Research Society; ISTH, International Society on Thrombosis and Haemostasis; THSNA, Thrombosis and Hemostasis Societies of North America; WFH, World Federation of Hemophilia.

Among the 105 included studies, 34 were clinical trials (single-arm trials, randomized controlled trials [RCTs], and non-RCTs) and 71 were observational studies (prospective and retrospective cohort studies) published between 2005 and 2025. The number of patients with hemophilia B ranged from 9 to 117 in clinical trials and 1 to 739 in observational studies. One clinical trial did not report the number of patients.

Studies enrolled patients of any age (8 clinical trials and 47 observational studies), patients <12 years (9 clinical trials and 6 observational studies), patients ≥12 years (13 clinical trials and 4 observational studies), and adults ≥18 years only (4 clinical trials and 14 observational studies). Most studies were multinational (26 clinical trials and 9 observational studies), 31 were conducted in Europe (all observational studies), 16 were conducted in North America (all observational studies), 10 were conducted in Asia (3 clinical trials and 7 observational studies), 2 observational studies were conducted in Australia, 1 clinical trial was conducted in Russia, 1 observational study was conducted in South America, and 10 studies did not report geography (4 clinical trials and 6 observational studies). Detailed patient characteristics are outlined in the Supplementary file.

Seventy-seven studies (24 clinical trials and 53 observational studies) were included in the meta-analysis. Of the 28 studies not included in the analyses, the reasons for exclusion were as follows: 7 studies did not report eligible outcome data; 16 studies reported the mean or median with no variance for rate outcomes; 3 reported data that were not comparable in a meta-analysis; 1 did not report the number of patients; and in 1 study, data were not stratified for patients who received prophylaxis vs on-demand therapy.

Funnel plot analyses were conducted to evaluate publication bias across outcomes in hemophilia B studies. Visual inspection indicated generally symmetrical distributions for AJBR, spontaneous bleeds, and zero treated bleeds, supported by Egger’s test results (*P* = .099, *P* = .780, and *P* = .123, respectively), suggesting minimal evidence of small-study effects. In contrast, ABR outcomes demonstrated notable asymmetry. Egger’s test was significant for observational ABR (treated and unspecified; *P* = .012) and clinical trial ABR (treated and unspecified; *P* = .031), indicating potential publication bias or heterogeneity. Clinical trial ABR (treated only) and spontaneous bleed outcomes showed nonsignificant Egger’s tests (*P* = .515 and *P* = .125), reducing concern for bias in these subsets. These findings highlight the need for cautious interpretation of ABR-related results, while other outcomes appear less affected by publication bias (Supplementary Figure 1).

### 3.2 Meta-analysis

#### 3.2.1 Annualized bleeding rate

Combined ABR values were slightly higher in clinical trials than in observational studies. Of 24 clinical trials in the meta-analysis, only 2 reported the total ABR, both of which were from the lead-in study/phase of gene therapy trials (4.19 and 4.41) [20,21]. Thirteen clinical trials including 547 patients reported treated ABR, and the combined mean was 2.23 (95% CI, 1.52-3.27) in the random-effects model and 2.56 (95% CI, 2.29-2.87) in the fixed-effect model (Figure 2; Supplementary Table 3). In 22 clinical trials, including 965 patients, the combined mean for treated and unspecified ABR was 2.25 (95% CI, 1.67-3.03) in the random-effects model and 2.74 (95% CI, 2.51-2.98) in the fixed-effect model (Figure 2; Supplementary Table 3).

**Figure 2:**
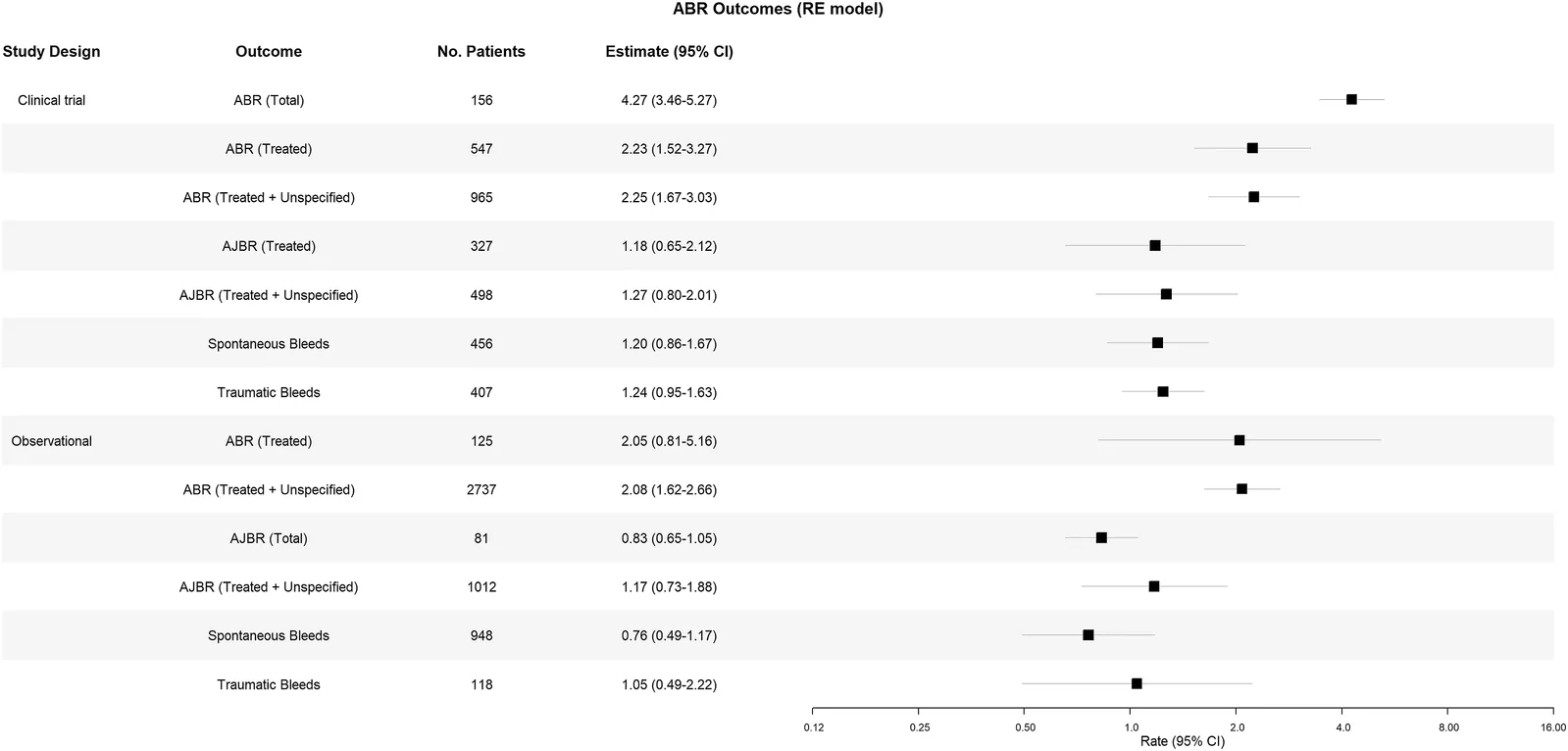
Annualized bleeding rate (ABR) outcomes for clinical trial and observational studies—random-effects model. AJBR, annualized joint bleeding rate.

In 53 observational studies included in the meta-analysis, no study specified total ABR. Three studies specified treated ABR, and the combined mean was 2.05 (95% CI, 0.81-5.16; 125 patients) in the random-effects model and 2.42 (95% CI, 1.93-3.03) in the fixed-effect model. In the analysis of 48 observational studies with 2737 patients, the combined mean for treated and unspecified ABR was 2.08 (95% CI, 1.62-2.66) in the random-effects model and 2.47 (95% CI, 2.37-2.58) in the fixed-effect model (Figure 2; Supplementary Table 3).

#### 3.2.2 Annualized joint bleeding rate

Clinical trials reported slightly higher AJBR than observational studies. In 6 clinical trials including 327 patients, the combined mean for treated AJBR was 1.18 (95% CI, 0.65-2.12) in the random-effects model and 1.45 (95% CI, 1.20-1.74) in the fixed-effect model. The combined mean treated and unspecified AJBR in clinical trials was 1.27 (95% CI, 0.80-2.01; 9 clinical trials, 498 patients) in the random-effects model and 1.58 (95% CI, 1.35-1.83) in the fixed-effect model (Figure 2; Supplementary Table 3).

Only 2 observational studies reported total AJBR with a combined mean of 0.83 (95% CI, 0.65-1.05; 81 patients) in both the random-effects model and fixed-effect model. No studies specified treated AJBR, whereas 18 studies including 1012 patients had a combined mean treated and unspecified AJBR of 1.17 (95% CI, 0.73-1.88) in the random-effects model and 1.34 (95% CI, 1.24-1.45) in the fixed-effect model (Figure 2; Supplementary Table 3).

#### 3.2.3 Spontaneous bleeds

The rate of spontaneous bleeds was higher in clinical trials compared with observational studies. In 10 clinical trials including 456 patients, the combined mean for spontaneous bleeds was 1.20 (95% CI, 0.86-1.67) in the random-effects model and 1.26 (95% CI, 1.07-1.49) in the fixed-effect model (Figure 2; Supplementary Table 3). Across 17 observational studies including 948 patients, the combined mean for spontaneous bleeds was 0.76 (95% CI, 0.49-1.17) in the random-effects model and 1.32 (95% CI, 1.21-1.44) in the fixed-effect model (Figure 2; Supplementary Table 3).

#### 3.2.4 Traumatic bleeding events

Rates of traumatic bleeds were similar across clinical trials and observational studies; however, analyses are based on a small number of observational studies. In 8 clinical trials including 407 patients, the combined mean for traumatic bleeds was 1.24 (95% CI, 0.95-1.63) in the random-effects model and 1.23 (95% CI, 1.05-1.43) in the fixed-effect model (Figure 2; Supplementary Table 3). Across 3 observational studies and 118 patients, the combined mean for traumatic bleeds was 1.05 (95% CI, 0.49-2.22) in the random-effects model and 0.88 (95% CI, 0.63-1.25) in the fixed-effect model (Figure 2).

#### 3.2.5 Percentage of patients with zero bleeds

The percentage of patients with zero treated and unspecified bleeding events was notably higher in observational studies compared with clinical trials; however, variability across study duration and study designs may influence these findings. Across 7 clinical trials including 267 patients with a median follow-up time of 27 months (range, 6-100; 1 study did not report a median follow-up time), the combined mean of percent zero bleeds (treated and unspecified) was 29.1% (95% CI, 23.8-34.6) in both the random-effects model and fixed-effect model (Figure 3; Supplementary Table 3). In 16 observational studies including 876 patients with a median follow-up time of 12.3 months (range, 8-51; 11 studies did not report a median follow-up time), the combined mean of percent zero bleeds (treated and unspecified) was 47.2% (95% CI, 34.4-60.2) in the random-effects model and 44.4 (95% CI, 41.2-47.7) in the fixed-effect model (Figure 4; Supplementary Table 3).

**Figure 3:**
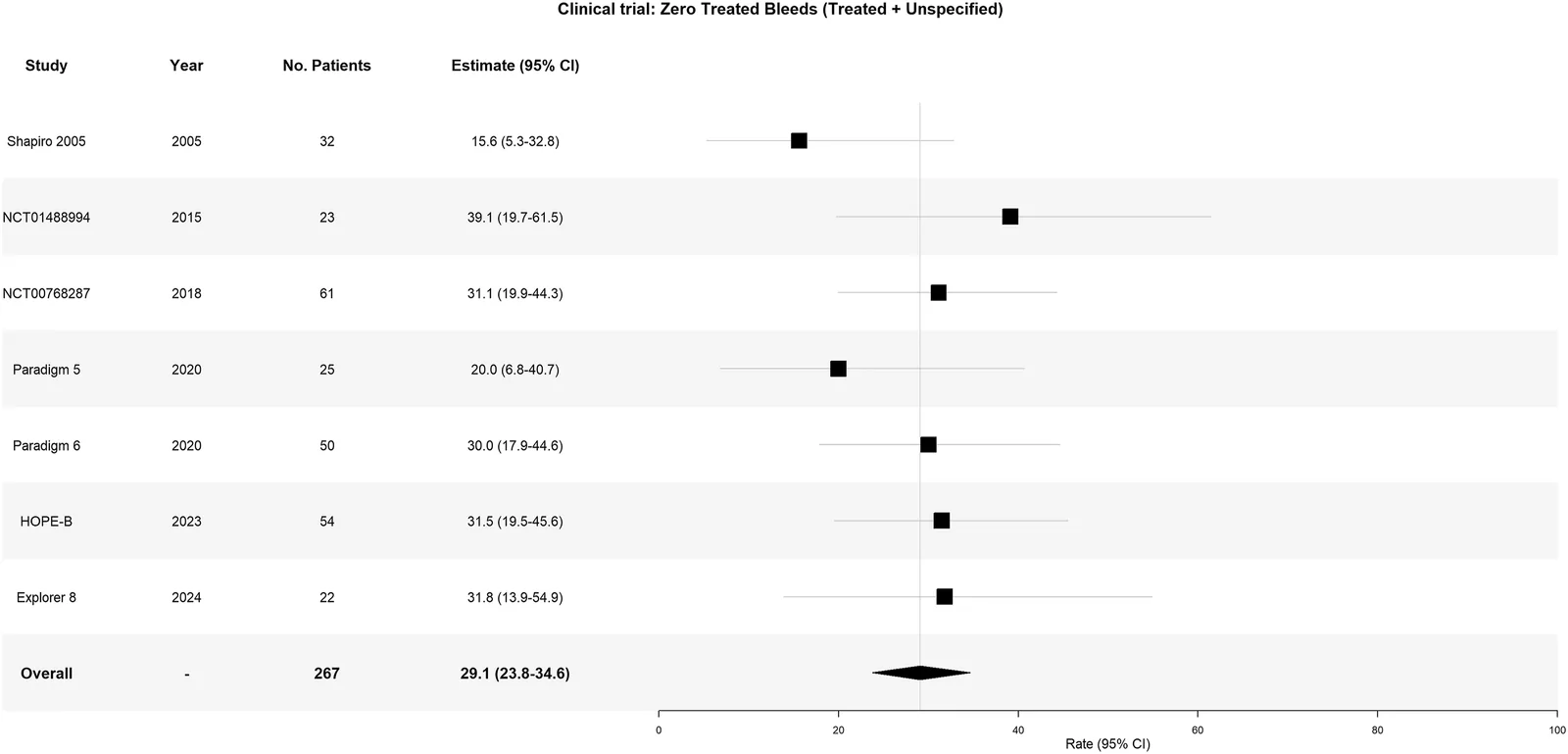
Percentage of patients with zero treated and unspecified bleeding events for clinical trials—random-effects model.

**Figure 4:**
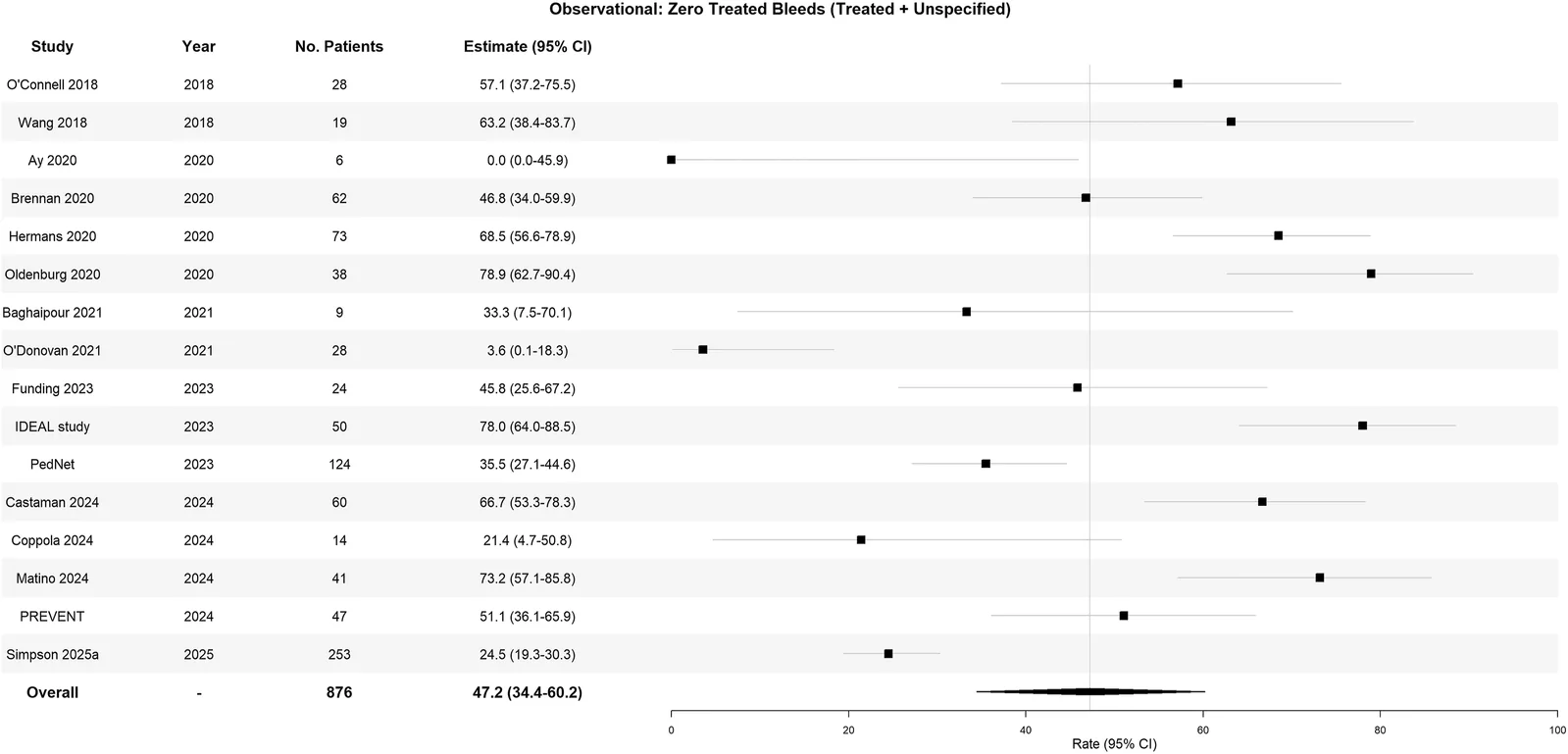
Percentage of patients with zero treated and unspecified bleeding events for observational studies—random-effects model.

### 3.3 Subgroup analyses for ABR

#### 3.3.1 Age

In clinical trials, patients ≥12 years had higher ABR estimates compared with observational studies, whereas among patients <12 years, ABR estimates were higher in observational studies. Among patients <12 years and ≥12 years and in clinical trials, the combined mean treated ABR was 0.82 (95% CI, 0.31-2.15; 3 clinical trials, 83 patients) and 2.92 (95% CI, 2.34-3.66; 9 clinical trials, 403 patients), respectively; the combined mean for treated and unspecified ABR among patients <12 years was 1.43 (95% CI, 0.72-2.82; 8 trials, 218 patients) and for ≥12 years it was 3.03 (95% CI, 2.52-3.65; 11 trials, 551 patients) (Supplementary Figure 2). There were an insufficient number of observational studies that stratified treated ABR by age; however, the combined mean for treated and unspecified ABR among patients <12 years and ≥12 years was 1.86 (95% CI, 0.72-4.78; 9 studies, 119 patients) and 1.88 (95% CI, 1.25-2.83; 18 studies, 1166 patients), respectively (Supplementary Figure 2).

#### 3.3.2 Follow-up duration

Overall, the highest bleeding rates were observed among clinical trials with <12 months of follow-up duration for treated ABR and treated and unspecified ABR. In clinical trials, the combined mean for treated ABR among patients with follow-up durations <12 months and ≥12 months was 2.97 (95% CI, 2.19-4.05; 5 clinical trials, 257 patients) and 1.73 (95% CI, 0.80-3.77; 6 clinical trials, 228 patients), respectively; the combined mean for treated and unspecified ABR was 2.64 (95% CI, 1.69-4.14; 10 clinical trials, 511 patients) and 1.92 (95% CI, 1.20-3.08; 10 clinical trials, 392 patients), respectively (Supplementary Figure 3).

There were an insufficient number of observational studies that reported treated ABR; however, the combined mean for treated and unspecified ABR with <12 months follow-up and ≥12 months follow-up was 2.02 (95% CI, 0.52-7.86; 4 studies, 210 patients) and 2.04 (95% CI, 1.59-2.63; 40 studies, 2338 patients), respectively (Supplementary Figure 3). Observational studies tended to report follow-up durations of ≥12 months or did not report the follow-up duration; therefore, most subgroup analyses of follow-up duration were not feasible.

#### 3.3.3 Treatment type

Generally, lower ABRs were observed in patients who received EHL compared with SHL treatment. The combined mean for treated ABR among patients treated with EHL and SHL in clinical trials was 1.37 (95% CI, 0.72-2.61; 6 clinical trials, 255 patients) and 3.58 (95% CI, 2.50-5.11; 2 clinical trials, 86 patients), respectively. However, this finding should be interpreted cautiously as the SHL estimate was based on only 2 clinical trials. The combined mean for treated and unspecified ABR was 1.50 (95% CI, 0.91-2.49; 8 clinical trials, 375 patients) and 2.61 (95% CI, 1.60-4.25; 9 clinical trials, 384 patients), respectively. In observational studies, the combined mean for treated and unspecified ABR among patients treated with EHL and SHL followed a similar trend and was 1.70 (95% CI, 1.31-2.22; 34 studies, 1878 patients) and 5.10 (95% CI, 3.49-7.45; 9 studies, 381 patients), respectively (Supplementary Figure 4).

#### 3.3.4 Date of publication

No substantial differences in ABR outcomes were observed by publication year, except for observational studies published before 2020 for treated ABR, which showed the highest rate (4.40, 95% CI, 3.10-6.25). However, this estimate should be interpreted with caution, as it was derived from one study. In clinical trials, treated ABR among publications before 2020 vs during 2020 and after was 2.47 (95% CI, 1.77-3.45; 6 clinical trials, 288 patients) and 2.00 (95% CI, 1.00-4.03; 7 clinical trials, 259 patients), respectively; treated and unspecified ABR among publications before 2020 vs during 2020 and after was 2.72 (95% CI, 2.14-3.46; 9 clinical trials, 459 patients) and 1.93 (95% CI, 1.18-3.14; 13 clinical trials, 506 patients), respectively. In observational studies, treated ABR among publications before 2020 vs during 2020 and after was 4.40 (95% CI, 3.10-6.25; 1 study, 22 patients) and 1.39 (95% CI, 0.55-3.53; 2 studies, 103 patients), respectively. However, these findings should be interpreted with caution given the limited evidence base. The combined mean for treated and unspecified ABR was 3.73 (95% CI, 2.91-4.78; 10 studies, 562 patients) and 1.76 (95% CI, 1.31-2.35; 38 studies, 2175 patients), respectively (Supplementary Figure 5). Additional results for the percentage of patients with zero treated and unspecified bleeding events for all subgroups are presented in the supplementary material (Supplementary Figure 6).

## 4 Discussion

This systematic review included 105 studies of patients with hemophilia B without inhibitors who received prophylactic factor replacement treatment, of which 24 clinical trials and 53 observational studies were included in the meta-analysis. Although progress has been made in the management of hemophilia B, the combined estimates from both clinical trials and observational studies indicated that mean treated ABR and mean treated and unspecified ABR remained >2 events per year (range, 2.05-2.25). These findings suggest that patients continue to experience bleeding episodes while receiving FIX replacement therapy, which may have implications for their overall quality of life.

Bleeding rates were generally higher in clinical trials compared with observational studies across most outcomes, likely reflecting differences in patient selection, monitoring intensity, and reporting quality. Clinical trials frequently enroll patients with more severe hemophilia or higher bleeding risk [22], whereas observational studies include broader real-world populations that may include patients with milder disease or are well-controlled on long-term prophylaxis [23]. A major concern is that observational studies are prone to poorer data quality and underreporting compared with clinical trials, which may distort bleeding rate estimates [24]. Unlike trials that employ active surveillance and rigorous reporting standards—capturing even minor events—routine care settings often fail to document all bleeds, especially less severe episodes, as bleeding events are often based on patient or physician reporting. These design differences may contribute to systematic underreporting of bleeding events in observational data, warranting caution when comparing bleed rates across real-world and clinical trial settings.

This issue is particularly important when interpreting total ABR, as no observational studies of factor replacement therapy included in this review reported bleeding outcomes that explicitly captured both treated and untreated events. Historically, studies have not consistently distinguished between treated ABR and total ABR. To avoid excluding these studies and potential outcome misclassification, ABR estimates that were not clearly defined as either treated or total were classified as “unspecified” and analyzed with treated ABR because they did not explicitly define bleeding outcomes and were therefore considered more likely to reflect treatment-based bleeding events rather than capturing both treated and untreated bleeds. Therefore, the estimates for treated and unspecified ABR should not be interpreted as representing total ABR. Although ABR is the primary efficacy measure for hemophilia research [2], variability in how bleeding events are defined, reported, and recorded can substantially influence the interpretation of ABR estimates [25]. These concerns are reflected in recommendations from the Scientific and Standardization Committee of the International Society on Thrombosis and Haemostasis, which emphasize standardized bleed definitions, reporting of clinically relevant total bleeds, clear documentation of treatment policies when using treated bleeds, and observation periods of at least 12 months for ABR estimation [26]. Greater standardization would improve comparability and interpretability of bleeding outcomes in future hemophilia research.

In subgroup analyses by age, the higher rates in patients ≥12 years may reflect accumulated joint damage, the presence of target joints, and the recruitment of individuals with higher bleeding risk into clinical trials. This is consistent with Manco-Johnson et al. [27], who reported that adults (≥20 years) with severe hemophilia B had significantly higher AJBR compared with younger patients (2-9 years) and identified age as an independent risk factor for increased bleeding. These findings support that younger patients may have lower ABRs because of less chronic joint damage and earlier stabilization on prophylaxis.

Subgroup analyses by follow-up duration showed higher bleeding rates in studies with <12 months follow-up than those with ≥12 months. This pattern is consistent with Keipert et al. [28], who reported that shorter follow-up periods (eg, 6 months) were associated with higher ABR and more variable ABR estimates, whereas longer periods (≥12 months) provide more stable values. This is due, in part, to inconsistent index date definitions. Additionally, shorter follow-up periods may inflate ABR estimates, as a small number of bleeds can have a disproportionate impact and positively skew the outcome, especially during early treatment adjustment [29].

Results of our meta-analysis are also consistent with other published reviews. Franchini et al. [30] compared prophylactic recombinant standard half-life (rSHL) FIX vs recombinant extended half-life (rEHL) FIX replacement therapy among patients with hemophilia B. Results showed that the pooled estimated mean ABR was lower in patients treated with rEHL (1.28 [95% CI, 0.91-1.66]), compared with rSHL (3.12 [95% CI, 2.63-3.62]) [30]. Similarly, a higher percentage of patients had zero-bleeding events when treated with rEHL (0.53 [95% CI, 0.37-0.69]), compared with rSHL (0.24 [95% CI, 0.14-0.39]) [30]. These results are well-aligned with the subgroup analysis by treatment in the current meta-analysis, where the combined mean treated ABR for patients treated with EHL and SHL was 1.37 (95% CI, 0.72-2.61) and 3.58 (95% CI, 2.50-5.11), respectively. These findings also align with the World Federation of Hemophilia 2020 Guidelines [2], which advocate for the use of EHL FIX products to support higher and sustained trough levels. These higher trough levels are expected to improve bleeding outcomes, including ABR, consistent with the lower ABRs observed in patients receiving EHL therapies in this review.

While the findings of this meta-analysis are generally consistent with prior reviews, our study provides a more granular synthesis of bleeding outcomes in hemophilia B by stratifying results according to study design, ABR definition, age, follow-up duration, treatment type, and publication period. This approach helps address key sources of heterogeneity that were not fully explored in prior reviews [30,31] and supports a more nuanced interpretation of bleeding burden among patients receiving prophylactic FIX replacement therapy. The inclusion of a broader range of subgroup analyses and both clinical trials and observational studies through 2025 also provides a more current and generalizable evidence base to inform the design and interpretation of future hemophilia studies.

This meta-analysis had some limitations. A key limitation of the available literature was that ABR definitions were not well reported, particularly with respect to whether bleeding outcomes reflected treated or total bleeds. This may have resulted in underestimation of bleeding burden when untreated bleeding events were not captured or explicitly reported. Additionally, some studies included a small number of patients with hemophilia B, and some subgroups in the analyses comprised only 1 or 2 studies, limiting interpretability. The subgroup analysis based on publication period should be interpreted with caution, as publication date was used as a proxy for study date and does not necessarily align with the dates over which the study was performed. The dataset also included global studies, and the regional rollout of EHLs varied. Therefore, any chosen date is arbitrary and may not capture the nuanced evolution of treatment types available across geographies. Interpretation of comparisons between SHL and EHL therapies is limited by differences in study era and design. SHL studies were conducted earlier and largely enrolled patients transitioning from on-demand regimens, likely representing a population with more severe disease. Additionally, some EHL studies included intrastudy comparisons with unequal ABR assessment periods, with shorter follow-up for SHL and longer follow-up for EHL, which may bias observed differences given higher ABR rates during follow-up periods of <12 months. Lastly, interpretation of the proportion of patients with zero-bleeding events is dependent on the duration of follow-up, as shorter observation periods may artificially inflate the apparent rate of zero events compared with studies with longer follow-up, complicating comparisons of efficacy between-study designs, treatment types, and regimens [25].

## 5 Conclusions

Significant advances in the treatment of hemophilia B are evident from this meta-analysis and have been translated into improved outcomes. Nevertheless, unmet needs remain, with combined zero-bleed proportions of 47.2% in observational studies and 29.1% in clinical trials, indicating that many patients continue to experience bleeding events. Interpretation of ABR outcomes is complicated by inconsistent definitions across studies, contributing to heterogeneity and limiting comparability. Additionally, higher ABR rates observed in clinical trials among patients ≥12 years likely reflect enrollment of individuals with more severe disease, whereas ABR rates in patients <12 years more closely aligned with real-world evidence. Lower ABR was associated with EHL therapies and longer follow-up (≥12 months) compared with SHL products and shorter follow-up. These findings should be interpreted cautiously given older study SHL study dates and the enrolment of patients largely from on-demand regimens, potentially reflecting more severe disease. Overall, variability in ABR definitions and follow-up duration limits reproducibility and generalizability, underscoring the need for standardized outcome measures and further research to inform clinical decision-making.
