# Supplementary material for: Bleeding events in people with hemophilia B on prophylactic factor IX treatment: a systematic review and meta-analysis
Source: Res Pract Thromb Haemost. 2026 Sep 9;10(7):106954. doi: 10.1016/j.rpth.2026.106954 (PMC13685439; doi:10.1016/j.rpth.2026.106954)
Supplement: Supplementary file 1 [file mmc1.docx]

SUPPLEMENTARY MATERIALS

# Table S1. PRISMA Checklist

| **Section and Topic** | **Item #** | **Checklist item** | **Location where item is reported** |
| --- | --- | --- | --- |
| **TITLE** | | |  |
| Title | 1 | Identify the report as a systematic review. | Title page |
| **ABSTRACT** | | |  |
| Abstract | 2 | See the PRISMA 2020 for Abstracts checklist. | 1 |
| **INTRODUCTION** | | |  |
| Rationale | 3 | Describe the rationale for the review in the context of existing knowledge. | 3-4 |
| Objectives | 4 | Provide an explicit statement of the objective(s) or question(s) the review addresses. | 4 |
| **METHODS** | | |  |
| Eligibility criteria | 5 | Specify the inclusion and exclusion criteria for the review and how studies were grouped for the syntheses. | 5-6 |
| Information sources | 6 | Specify all databases, registers, websites, organisations, reference lists and other sources searched or consulted to identify studies. Specify the date when each source was last searched or consulted. | 5-6 |
| Search strategy | 7 | Present the full search strategies for all databases, registers and websites, including any filters and limits used. | Suppl Table S2 |
| Selection process | 8 | Specify the methods used to decide whether a study met the inclusion criteria of the review, including how many reviewers screened each record and each report retrieved, whether they worked independently, and if applicable, details of automation tools used in the process. | 5-6 |
| Data collection process | 9 | Specify the methods used to collect data from reports, including how many reviewers collected data from each report, whether they worked independently, any processes for obtaining or confirming data from study investigators, and if applicable, details of automation tools used in the process. | 5-6 |
| Data items | 10a | List and define all outcomes for which data were sought. Specify whether all results that were compatible with each outcome domain in each study were sought (e.g. for all measures, time points, analyses), and if not, the methods used to decide which results to collect. | 6 |
|  | 10b | List and define all other variables for which data were sought (e.g. participant and intervention characteristics, funding sources). Describe any assumptions made about any missing or unclear information. | 6 |
| Study risk of bias assessment | 11 | Specify the methods used to assess risk of bias in the included studies, including details of the tool(s) used, how many reviewers assessed each study and whether they worked independently, and if applicable, details of automation tools used in the process. | 8 |
| Effect measures | 12 | Specify for each outcome the effect measure(s) (e.g. risk ratio, mean difference) used in the synthesis or presentation of results. | 6-8 |
| Synthesis methods | 13a | Describe the processes used to decide which studies were eligible for each synthesis (e.g. tabulating the study intervention characteristics and comparing against the planned groups for each synthesis (item #5)). | 6-8 |
|  | 13b | Describe any methods required to prepare the data for presentation or synthesis, such as handling of missing summary statistics, or data conversions. | 7-8 |
|  | 13c | Describe any methods used to tabulate or visually display results of individual studies and syntheses. | 7-8 |
|  | 13d | Describe any methods used to synthesize results and provide a rationale for the choice(s). If meta-analysis was performed, describe the model(s), method(s) to identify the presence and extent of statistical heterogeneity, and software package(s) used. | 6-7 |
|  | 13e | Describe any methods used to explore possible causes of heterogeneity among study results (e.g. subgroup analysis, meta-regression). | 6-7 |
|  | 13f | Describe any sensitivity analyses conducted to assess robustness of the synthesized results. | 7-8 |
| Reporting bias assessment | 14 | Describe any methods used to assess risk of bias due to missing results in a synthesis (arising from reporting biases). | 8 |
| Certainty assessment | 15 | Describe any methods used to assess certainty (or confidence) in the body of evidence for an outcome. | 7-8 |
| **RESULTS** | | |  |
| Study selection | 16a | Describe the results of the search and selection process, from the number of records identified in the search to the number of studies included in the review, ideally using a flow diagram. | 8-9 |
|  | 16b | Cite studies that might appear to meet the inclusion criteria, but which were excluded, and explain why they were excluded. | 9-10 |
| Study characteristics | 17 | Cite each included study and present its characteristics. | 8-10, Suppl excel |
| Risk of bias in studies | 18 | Present assessments of risk of bias for each included study. | 10, Suppl |
| Results of individual studies | 19 | For all outcomes, present, for each study: (a) summary statistics for each group (where appropriate) and (b) an effect estimate and its precision (e.g. confidence/credible interval), ideally using structured tables or plots. | 10-15, Suppl |
| Results of syntheses | 20a | For each synthesis, briefly summarise the characteristics and risk of bias among contributing studies. | 8-9 |
|  | 20b | Present results of all statistical syntheses conducted. If meta-analysis was done, present for each the summary estimate and its precision (e.g. confidence/credible interval) and measures of statistical heterogeneity. If comparing groups, describe the direction of the effect. | 10-15, Suppl |
|  | 20c | Present results of all investigations of possible causes of heterogeneity among study results. | 10-15, Suppl |
|  | 20d | Present results of all sensitivity analyses conducted to assess the robustness of the synthesized results. | 13-15, Suppl |
| Reporting biases | 21 | Present assessments of risk of bias due to missing results (arising from reporting biases) for each synthesis assessed. | Suppl |
| Certainty of evidence | 22 | Present assessments of certainty (or confidence) in the body of evidence for each outcome assessed. | 10-15, Suppl |
| **DISCUSSION** | | |  |
| Discussion | 23a | Provide a general interpretation of the results in the context of other evidence. | 16-20 |
|  | 23b | Discuss any limitations of the evidence included in the review. | 19-20 |
|  | 23c | Discuss any limitations of the review processes used. | 19-20 |
|  | 23d | Discuss implications of the results for practice, policy, and future research. | 20-21 |
| **OTHER INFORMATION** | | |  |
| Registration and protocol | 24a | Provide registration information for the review, including register name and registration number, or state that the review was not registered. | 4 |
|  | 24b | Indicate where the review protocol can be accessed, or state that a protocol was not prepared. | 4 |
|  | 24c | Describe and explain any amendments to information provided at registration or in the protocol. | NA |
| Support | 25 | Describe sources of financial or non-financial support for the review, and the role of the funders or sponsors in the review. | 22-23 |
| Competing interests | 26 | Declare any competing interests of review authors. | 22-23 |
| Availability of data, code and other materials | 27 | Report which of the following are publicly available and where they can be found: template data collection forms; data extracted from included studies; data used for all analyses; analytic code; any other materials used in the review. | NA |

# Table S2. Literature review search strategy

| **#** | **Searches** | **Results from January 29, 2025** |
| --- | --- | --- |
| **1** | hemophilia b/ or (hemophilia b$ or haemophilia b$ or F9 deficienc$ or christmas disease or factor ix deficienc$ or antihemophilic factor b or antihaemophilic factor b or hemophilic b or haemophilic b or factor 9 deficienc$).ti,ab. | 18,380 |
| **2** | Randomized controlled trials as Topic/ or Randomized controlled trial/ or Random allocation/ or Double blind method/ or Single blind method/ or Clinical trial/ or exp Clinical Trials as Topic/ or ((clinic$ adj trial$1) or ((singl$ or doubl$ or treb$ or tripl$) adj (blind$3 or mask$3))).tw. or Placebos/ or Placebo$.tw. or Randomly allocated.tw. or (allocated adj2 random).tw. or (single arm trial or singl* or single-arm or cross over or cross-over).tw. | 10,102,236 |
| **3** | exp registries/ or (registry or registries or register$1 or disease regist$).kw. or (registries or registry or disease regist$3 or register$1 or national regist$ or h?emophilia regist$).ti,ab,kw. or (h?emophilia adj2 database$).ti,ab. | 870,597 |
| **4** | Databases, Factual/ or exp Electronic Health Records/ or insurance claim review/ or (real-world or real life).ti. or ((real world or real life) adj3 (data$ or setting or cohort$ or stud$ or experience$ or result$)).ti,ab. or (Database analys#s or database stud$ or claims data).ti,ab,kw. or (data adj (report$ or data system$1)).ti,ab. or (chart review or medical record review or (review adj5 patient records)).ti,ab. or (Claims data or health care database$ or claims database$ or health insurance database$ or health insurance claim$ or health insurance research database$).ti,ab,kw. or (Health insurance adj5 database$).ti,ab. | 809,177 |
| **5** | Cross-sectional studies/ or case control studies/ or exp cohort studies/ or Follow-Up Studies/ or Retrospective Studies/ or Controlled Before-After Studies/ or observational studies as topic/ or observational study/ or pragmatic clinical trial/ or Adaptive Clinical Trial/ or Comparative Study/ or (Long-term or observational or cohort? or longitudinal or prospective or retrospective or comparative or case-control or followup or follow-up or comparative or cross-sectional).ti. or (cohort analys$ or cohort stud$ or longitudinal stud$ or chart review$ or medical record review$ or observational stud$ or retrospective analys$ or retrospective chart review? or retrospective clinical stud$ or retrospective cohort$ or retrospective stud$ or retrospective database$ or retrospective observational$ or case control stud$ or case control setting or nested case control stud$ or matched case-control stud$ or pragmatic stud$ or followup stud$ or follow-up stud$ or Follow-up or followup or open label extension stud$3 or cross-sectional stud$ or cross-sectional survey).kf. or ((Cohort or Case control or longitudinal or retrospective or pragmatic or adaptive) adj2 (study or studies)).ti,ab. or ((observational or prospective) adj3 (study or studies or cohort$1 or data$ or clinical or analys#s)).ti,ab. or ((Population-based or nationwide or community based) adj2 (study or studies or cohort$1 or survey$1)).ti,ab. or (pre-post intervention stud$3 or pre-post stud$3).ti,ab. or Health Surveys/ or health care surveys/ or (health survey$1 or health care survey$1 or medical care survey$1 or treatment survey$1 or national survey$ or survey$1).ti,kw. | 14,144,188 |
| **6** | 2 or 3 or 4 or 5 | 22,074,682 |
| **7** | exp Books/ or exp Case Reports/ or exp Models, Theoretical/ or (letter or editorial or erratum or note or short survey).pt. | 10,395,641 |
| **8** | animal cell/ or animal cell.mp. or animal experiment/ or animal experiment.mp. or animal model/ or animal model.mp. or cancer cell culture/ or cancer cell culture.mp. or human cell/ or human cell.mp. or human tissue/ or human tissue.mp. or in vitro study.mp. or nonhuman/ or nonhuman.mp. or exp biological model/ or biological model.mp. or exp cell culture/ or cell culture.mp. or diagnostic test accuracy study/ or diagnostic test accuracy study.mp. | 13,870,593 |
| **9** | 7 or 8 | 22,845,928 |
| **10** | (1 and 6) not 9 | 5,941 |
| **11** | limit 10 to English only  MEDLINE = 1,799  Embase = 3,722  CENTRAL = 163 | 5,684 |
| **12** | Remove duplicates in OVID | 4,583 |
| **13** | Remove duplicates in Distiller | 4,553 |

# Table S3. Fixed-effect model results for the overall population

| **Study design** | **Outcome** | **No. studies** | **No. patients** | **pred** | **ci.lb** | **ci.ub** | **i2** | **P value** |
| --- | --- | --- | --- | --- | --- | --- | --- | --- |
| Clinical trial | ABR (Total) | 2 | 156 | 4.27 | 3.46 | 5.27 | 0.0 | 0.82 |
| Clinical trial | ABR (Treated) | 13 | 547 | 2.56 | 2.29 | 2.87 | 86.59 | < 0.001 |
| Clinical trial | ABR (Treated + Unspecified) | 22 | 965 | 2.74 | 2.51 | 2.98 | 86.42 | < 0.001 |
| Clinical trial | ABR (Treated + Unspecified) | 22 | 965 | 2.25 | 1.67 | 3.03 | 86.42 | < 0.001 |
| Clinical trial | AJBR (Treated) | 6 | 327 | 1.45 | 1.20 | 1.74 | 88.14 | < 0.001 |
| Clinical trial | AJBR (Treated + Unspecified) | 9 | 498 | 1.58 | 1.35 | 1.83 | 85.88 | < 0.001 |
| Clinical trial | AJBR (Treated + Unspecified) | 9 | 498 | 1.27 | 0.80 | 2.01 | 85.88 | < 0.001 |
| Clinical trial | Spontaneous Bleeds | 10 | 456 | 1.26 | 1.07 | 1.49 | 69.14 | < 0.001 |
| Clinical trial | Traumatic Bleeds | 8 | 407 | 1.23 | 1.05 | 1.43 | 66.52 | 0.004 |
| Clinical trial | Zero Treated Bleeds (Treated + Unspecified) | 7 | 267 | 0.29 | 0.24 | 0.35 | 0.0 | 0.488 |
| Observational | ABR (Treated) | 3 | 125 | 2.42 | 1.93 | 3.03 | 92.87 | < 0.001 |
| Observational | ABR (Treated + Unspecified) | 48 | 2737 | 2.47 | 2.37 | 2.58 | 95.56 | < 0.001 |
| Observational | AJBR (Total) | 2 | 81 | 0.83 | 0.65 | 1.05 | 0.0 | 0.571 |
| Observational | AJBR (Treated + Unspecified) | 18 | 1012 | 1.34 | 1.24 | 1.45 | 95.84 | < 0.001 |
| Observational | Spontaneous Bleeds | 17 | 948 | 1.32 | 1.21 | 1.44 | 96.50 | < 0.001 |
| Observational | Traumatic Bleeds | 3 | 118 | 0.88 | 0.63 | 1.25 | 61.45 | 0.075 |
| Observational | Zero Treated Bleeds (Treated + Unspecified) | 16 | 876 | 0.44 | 0.41 | 0.48 | 91.72 | < 0.001 |

Abbreviations: ABR = annualized bleeding rate; AJBR= annualized joint bleeding rate; CI= confidence interval; lb= lower bound; ub= upper bound

# Figure S1. Funnel plots

A. Treated ABR in clinical trials

B. Treated + unspecified ABR in clinical trials
C. Treated + unspecified ABR in observational studies

D. Treated + unspecified AJBR in observational studies

E. Spontaneous bleeds in clinical trials
F. Spontaneous bleeding events in clinical trials

G. Spontaneous bleeding events in observational studies

H. Percentage of patients with zero treated + unspecified bleeding events in observational studies

# Figure S2. ABR outcomes, stratified by age group (12+ years old, <12 years old)

ABR = annualized bleeding rate; AJBR = annualized joint bleeding rate; CI = confidence interval

# Figure S3. ABR outcomes, stratified by follow-up duration (12+ months, <12 months)

ABR = annualized bleeding rate; AJBR = annualized joint bleeding rate; CI = confidence interval

# Figure S4. ABR outcomes, stratified by treatment type (EHL, SHL)

ABR = annualized bleeding rate; AJBR = annualized joint bleeding rate; CI = confidence interval; EHL = extended half-life factor replacement therapy; SHL = standard half-life factor replacement therapy; Tx = treatment

# Figure S5. ABR outcomes, stratified by year of publication (during and after 2020, before 2020)

ABR = annualized bleeding rate; AJBR = annualized joint bleeding rate; CI = confidence interval

# Figure S6. Percentage of patients with zero treated + unspecified bleeding events by subgroup

ABR = annualized bleeding rate; AJBR = annualized joint bleeding rate; CI = confidence interval; EHL = extended half-life factor replacement therapy; SHL = standard half-life factor replacement therapy; Tx = treatment
